# The Choice of a Hospital Secretary

**Published:** 1911-04-15

**Authors:** 


### THE CHOICE OF A HOSPITAL SECRETARY.

A Hospital Secretary writes to us as follows :—May a hospital secretary be forgiven if he expresses disappointment at the tone of your note in last week's issue of The Hospital? You do not even urge the importance of originality on the part of a man who would be successful as a hospital secretary. It seems to me that no individual can hope to serve his hospital well in the capacity of secretary unless, in the first place, he has been trained to the work; unless, too, he has a knowledge of the world and its affairs; unless he is an acute student of human nature; unless he has about him something quite akin to the qualities which go to make a prosperous common showman; unless he has the knack of making other people work and of choosing the right sort of person as his helper; unless he takes a pride in his work and is never ashamed to hear himself associated with it, even when at a private dinner table; unless, in short, he is a wholly exceptional and an accomplished fellow.

